# Association of exposure to multiple volatile organic compounds with ultrasound-defined hepatic steatosis and fibrosis in the adult US population: NHANES 2017–2020

**DOI:** 10.3389/fpubh.2024.1437519

**Published:** 2025-01-17

**Authors:** Wentao Shao, Pan Gong, Qihan Wang, Fan Ding, Weiyi Shen, Hongchao Zhang, Anhua Huang, Chengyu Liu

**Affiliations:** ^1^School of Instrument Science and Engineering, Southeast University, Nanjing, China; ^2^Center of Gallstone Disease, Shanghai East Hospital, Tongji University School of Medicine, Shanghai, China; ^3^Hongkou District Center for Disease Control and Prevention (Hongkou District Institute of Health Supervision), Shanghai, China

**Keywords:** volatile organic compound, urinary metabolites, hepatic steatosis, liver fibrosis, NHANES

## Abstract

**Objective:**

Volatile organic compounds (VOCs) are pervasive environmental pollutants known to impact human health, but their role in liver steatosis or fibrosis is not fully understood. This study investigates the association of urinary VOC mixtures with the risk of liver steatosis and fibrosis in U.S. adult population.

**Methods:**

Data of 1854 adults from the National Health and Nutrition Examination Survey (NHANES) from 2017.01 to 2020.03 were collected. Vibration Controlled Transient Elastography (VCTE) assessed hepatic steatosis and liver fibrosis via the controlled attenuation parameter (CAP) and liver stiffness measurement (LSM), respectively. The study examined the relationship between urinary exposure biomarkers for 20 VOCs and liver health outcomes using multivariate logistic regression and Bayesian Kernel Machine Regression (BKMR) to evaluate the effects of both individual and mixed VOC exposures.

**Results:**

Multivariate logistic regression analysis revealed that exposure biomarkers for acrolein and crotonaldehyde were positively associated with hepatic steatosis. Conversely, biomarkers for styrene, ethylbenzene, and propylene oxide were negatively associated with hepatic steatosis. Furthermore, biomarkers for 1,3-butadiene and xylene were positively associated with liver fibrosis, while ethylbenzene was negatively associated with this condition. BKMR analysis identified a significant positive joint effect of VOC biomarkers on CAP. Notably, when other VOC-EBs were held at median levels, biomarkers for acrolein and 1,3-butadiene exhibited linear correlations with Ln CAP and hepatic Ln LSM, respectively.

**Conclusion:**

The study highlights the potential hepatotoxic effects of VOC mixtures, particularly noting the roles of acrolein and 1,3-butadiene in exacerbating liver steatosis and fibrosis. These findings advocate for further research to explore the mechanistic pathways and conduct longitudinal studies to establish causality and enhance understanding of VOCs’ impact on liver health.

## 1 Introduction

Non-alcoholic fatty liver disease (NAFLD) affects approximately 25% of the adult population and has become the most prevalent chronic liver disorder (1). By 2030, it is projected that NAFLD will affect 33.5% of the adult population (2). NAFLD encompasses a disease continuum related to metabolic dysfunction, ranging from steatosis to steatohepatitis, fibrosis, cirrhosis, and eventually hepatocellular carcinoma (1, 3). Although the specific pathogenic factors have not been fully elucidated, it is clear that genetic, epigenetic, and environmental factors influence liver steatosis and fibrosis progression (4). Recent evidence suggests that persistent exposure to certain environmental contaminants can initiate and promote the pathogenesis of NAFLD (5). Elucidating how these environmental contaminants, particularly volatile organic compounds (VOCs), either independently or in combination, affect hepatic steatosis and fibrosis is critical for disease prevention.

VOCs are among the most common environmental pollutants, originating from a variety of anthropogenic and natural sources, including cigarette smoke (6), vehicular exhaust (7), biomass burning, and industrial emissions (8). The United States Environmental Protection Agency (US EPA) has classified VOCs such as toluene, xylene, styrene, propylene oxide, 1,3-butadiene, vinyl chloride, trichloroethylene, tetrachloroethylene, acrylamide, acrylonitrile, acrolein, and carbon disulfide as hazardous air pollutants (9). Human exposure to VOCs through inhalation (the main exposure route), ingestion, and dermal contact is ubiquitous in daily life (10). Exposure to VOCs is associated with increased risks of leukemia, cancer, respiratory illnesses, birth defects, and neurocognitive impairment in humans (8). Tobacco smoking, a major source of VOC exposure, has been linked to sarcopenia, a condition characterized by reduced skeletal muscle mass and strength. Recent studies have demonstrated the association between smoking and muscle health, including reduced handgrip strength (11) and impaired respiratory muscle function (12). Sarcopenia, which is prevalent in advanced liver disease, has also been identified as a significant comorbidity in aging populations, with smoking serving as a key contributing factor (13). Additionally, sarcopenia is emerging as a therapeutic target, given its shared pathophysiology across multiple chronic diseases (14). These findings underscore the importance of considering smoking and VOC exposure as contributors to both sarcopenia and liver-related outcomes.

As the central hub of xenobiotic metabolism, including VOCs, the liver is a general target for the toxicity of environmental chemicals (15, 16). After exposure, VOCs quickly reach the liver via systemic circulation and are metabolized by hepatic cytochrome P450 (CYP450) enzymes (17, 18). In the liver, VOCs are transformed into water-soluble metabolites that are subsequently excreted in urine. Due to their specificity and longer half-lives in the human body compared to their parent compounds, urinary VOC metabolites may serve as reliable biomarkers of exposure (VOC-EBs) (19). Previous studies have demonstrated that workers occupationally exposed to VOCs can develop liver injury (20, 21). The scientific plausibility of VOCs influencing liver health and function is also supported by animal studies (22). However, most studies have primarily consisted of vulnerable or occupational populations and were usually based on recognized hazardous materials. Recent epidemiological studies conducted among 663 United States adults reported that metabolites of residential VOCs were positively associated with alkaline phosphatase (ALP), a biomarker for cholestatic injury (23). Another study involving 3,950 Canadian adults found that certain compounds in the benzene series were associated with poor liver function parameters (24).

Moreover, the association between VOCs and both NAFLD and liver fibrosis in humans remains unclear. A recent epidemiological study illustrated the relationship between VOC exposure and NAFLD, where NAFLD was defined using the US fatty liver index (USFLI) and the hepatic steatosis index (HSI), based on 12 serum markers (25). However, serum scores (HSI and FLI) performed poorly in detecting NAFLD and grading steatosis (26). These serum biomarkers can appear normal in patients with NAFLD and are influenced by comorbid conditions, potentially lacking sensitivity in defining NAFLD (26, 27). Liver vibration-controlled transient elastography (VCTE) using FibroScan, known for its high sensitivity and specificity, can directly assess hepatic steatosis and liver fibrosis via the controlled attenuation parameter (CAP) and liver stiffness measurement (LSM), respectively (1, 26–28). In the 2017–2018 survey cycle, for the first time, NHANES employed VCTE to measure CAP, indicating liver steatosis, and LSM, indicating liver fibrosis.

Thus, this study aims to examine the cross-sectional association between urinary VOC-EB levels and the prevalence of liver steatosis (measured by median CAP) and liver fibrosis (quantified as liver stiffness) detected by VCTE in NHANES participants from 2017 to 2020.

## 2 Materials and methods

### 2.1 Data source and study population

The NHANES is a national, multi-year, population-based, cross-sectional study conducted by the US National Center for Health Statistics (Centers for Disease Control and Prevention, Atlanta, GA, United States). Approval for the NHANES was granted by the National Center for Health Statistics Research Ethics Review Board, ensuring a representative sample of the non-institutionalized civilian US population. Liver steatosis and hepatic fibrosis were assessed using VCTE exclusively during the 2017.01–2020.03 cycle of NHANES; thus, this study is based on the dataset from this specific cycle.

**Figure 1:**
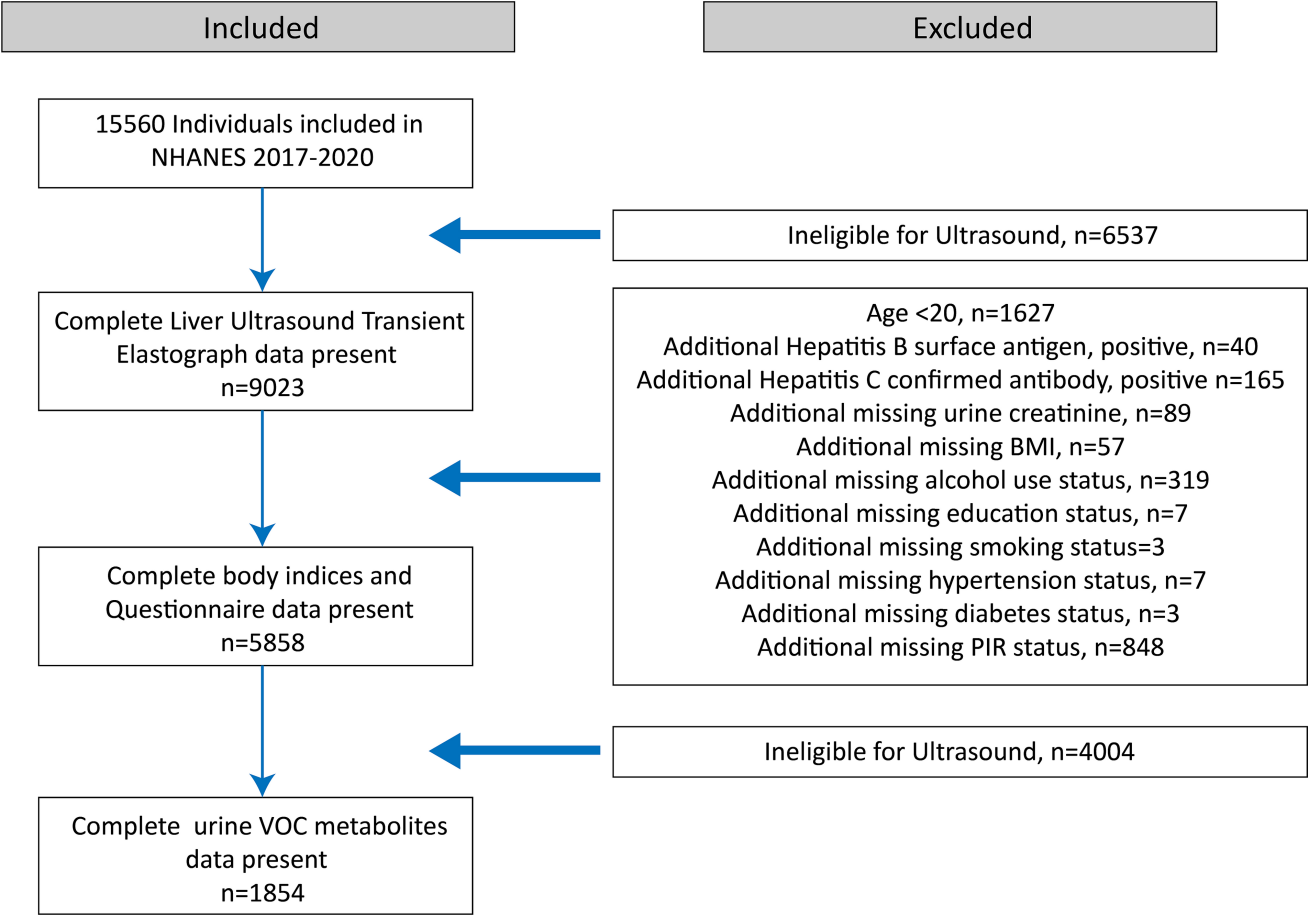
Flowchart illustrating the selection of the study population (*N* = 1854) for the final analysis, after applying exclusion criteria. Data were derived from the National Health and Nutrition Examination Survey (NHANES) conducted in the United States from 2017 to 2020.

A total of 15,560 eligible participants aged 6 years and older were included. Initially, participants with unavailable or incomplete VCTE exams were excluded (*n* = 6,537). Subsequently, we excluded participants younger than 20 years (*n* = 1,627); those with positive HBV surface antigens (*n* = 40), confirmed HCV antibodies (*n* = 165), or incomplete body indices and questionnaire data (*n* = 1,333). Further exclusions were made for participants lacking data on urine VOC metabolites (*n* = 4,004). Ultimately, 1,854 participants with complete data were enrolled (Figure 1).

### 2.2 Quantification of urine VOCs metabolites

Urine specimens were processed, stored, and shipped to the Division of Laboratory Sciences at the National Center for Environmental Health, Centers for Disease Control and Prevention, for analysis. Measurement of VOC metabolites was performed using ultra-performance liquid chromatography coupled with electrospray tandem mass spectrometry (UPLC-ESI/MSMS), as previously described (19). Twenty-one VOC metabolites were quantified in urine, including N-ace-S-(3,4-dihidxybutl)-L-cys (DHBMA), N-A-S-(4-hydrxy-2-butenyl)-L-cys (MHBMA3), 2-methylhippuric acid (2MHA), 3-methipurc acd + 4-methipurc acd (3MHA +4MHA), N-ace-S-(2-carbxyethyl)-L-cys (CEMA), N-ace-S-(3-hydroxypropyl)-L-cys (3HPMA), N-ace-S-(2-carbamoylethyl)-L-cys (AAMA), N-ac-S-(2-carbmo-2-hydxel)-L-cys (GAMA), mandelic acid (MA), phenylglyoxylic acid (PGA), N-ace-S-(1-cyano-2-hydroxyethyl)-L-cys (CHEMA), N-ace-S-(2-cyanoethyl)-L-cys (CEMA), N-ace-S-(2-hydroxyethyl)-L-cys (2HEMA), N-ace-S-(4-hydroxy-2-methyl-2-buten-1-yl)-L-cys (IPMA3), N-ace-S-(N-methlcarbamoyl)-L-cys (AMCC), 2-aminothiazoline-4-carboxylic acid (ATCA), N-ace -S-(benzyl)-L-cys (BMA), N-ace-S-(n-propyl)-L-cys (BPMA), N-ace-S-(2-hydroxypropyl)-L-cys (2HPMA), N-ace-S-(3-hydrxprpl-1-metl)-L-cys (HPMMA), and 2-Thioxothiazolidine-4-carboxylic acid (TTCA). As indicated by previous studies (17, 23, 25), when VOCs possess two or more metabolites, the levels of these metabolites are summed to represent the total exposure to the parent VOCs in this study, including ΣUBUM for 1,3-butadiene (DHBMA+MHBMA3), ΣUXM for xylene (2MHA + 3MHA + 4MHA), ΣUACLM for acrolein (CEMA+3HPMA), ΣUAAM for acrylamide (AAMA+ GAMA), ΣUSEBM for styrene and ethylbenzene (MA+PGA), and ΣUACLNM for acrylonitrile (CHEMA+ CEMA+2HEMA). TTCA was excluded from this study to maintain adequate statistical power, as it is the only metabolite of carbon disulfide with a detectable rate below 60%. Values below the lower limit of detection (LOD) for each metabolite were replaced by LOD/√2 (19). Finally, 13 urinary VOC-EBs were included in this analysis. Supplementary Table S1 lists 20 VOC metabolites, their parent compounds, and detectable rates.

### 2.3 Assessment of liver steatosis and fibrosis outcomes

CAP and LSM, indicators of liver steatosis and fibrosis respectively, were measured using VCTE. Trained technicians conducted VCTE assessments using a FibroScan model 502 V2 Touch (Echosens, Paris, France) equipped with either a medium (M) or extra-large (XL) probe. The medium probe was initially used. An XL probe was utilized if recommended by the manufacturer’s instructions. Examinations were deemed reliable if participants had fasted for at least 3 h before the exam, at least 10 complete stiffness measures were obtained, and the liver stiffness interquartile range to median LSM ratio was less than 30%. Detailed procedures are available in the Liver Ultrasound Transient Elastography Procedures Manual. Liver steatosis was defined as a CAP of ≥274 dB/m, a threshold demonstrating 90% sensitivity in identifying NAFLD (29). A threshold of LSM ≥8 kPa was established for liver fibrosis.

### 2.4 Assessment of covariates

Potential confounders associated with levels of VOC-EBs and liver steatosis/fibrosis, derived from NHANES questionnaires, examinations, and laboratory data, included gender, age, race-ethnicity, education level, smoking status, alcohol use, diabetes, hypertension, physical activity, and PIR (poverty income ratio). Given that the liver regulates energy homeostasis in a sex-dependent manner, we have included sex as one of the covariates. Covariate categories included: gender (male or female), race-ethnicity (Mexican American, Hispanic, non-Hispanic white, non-Hispanic black, or other), education level (high school or less, college, graduate or higher), smoking status (never: <100 cigarettes in lifetime; former: >100 cigarettes in lifetime and currently not smoking; current: >100 cigarettes in lifetime and smokes every day or occasionally), alcohol use (30) (never drinkers; low-moderate drinkers: ≤2 drinks per day on average for men and ≤1 drink per day on average for women, on days alcohol was consumed during the past year; heavy drinkers: >2 drinks per day on average for men and >1 drink per day on average for women, on days alcohol was consumed during the past year), diabetes (defined by an FPG level ≥ 7.0 mmol/L, an HbA1c ≥ 6.5%, or a self-reported history of diagnosis by a physician), overweight/obesity (BMI ≥ 25 kg/m^2^), hypertension (defined as systolic blood pressure ≥ 140 mmHg, diastolic blood pressure ≥ 90 mmHg, or a self-reported history of hypertension diagnosed by a physician), and physical activity (identified as having regular physical activity if they engaged in vigorous or moderate recreational activities). Additionally, to control for the urinary dilution effect of spot urine samples, urinary creatinine levels were adjusted for in all models as a covariate.

### 2.5 Statistical analysis

Descriptive statistics for continuous predictors, such as age, were obtained by calculating the mean value and standard deviation. Descriptive statistics for categorical variables were determined by calculating the number and frequency distributions for factors including gender, race, education, smoking status, alcohol use, diabetes, overweight/obesity, hypertension, physical activity, and PIR. We adjusted urinary VOC-EB concentrations for creatinine to minimize the effects of urine dilution. Although samples in the NHANES survey were weighted to reduce selection bias across subgroups based on age, sex, and ethnicity, we utilized unweighted estimations in our regression models, as the variables used for sample weighting were already incorporated in our study (31).

Initially, Pearson correlation coefficients were calculated between pairs of creatinine-adjusted VOC-EBs. These coefficients were categorized as weak (*r* ≤ 0.3), medium (0.3 < *r* ≤ 0.8), and strong (*r* > 0.8).

Secondly, we utilized multiple logistic regression models to evaluate the odds ratios (OR) with 95% confidence intervals (CIs) for the relationship between creatinine-adjusted VOC-EBs and liver steatosis and fibrosis. Creatinine-adjusted VOC-EB concentrations were categorized into quartiles, with the lowest quartile (Q1) serving as the reference group. The logistic regression analysis was adjusted for age, gender, race, obesity, diabetes, hypertension, smoking status, alcohol use, physical activity, education level, and poverty income ratio (PIR). Given that energy homeostasis exhibits sexual dimorphic traits and fatty liver diseases exhibit a strong sexual bias (32, 33), we constructed separate regression models for males and females.

Subsequently, the Bayesian kernel machine regression (BKMR) model, a non-parametric Bayesian variable selection framework, was employed to assess the joint effects of creatinine-adjusted VOC-EBs on liver steatosis and fibrosis (31). Data for CAP and LSM were transformed to natural logarithms to achieve a normal distribution. The BKMR model estimates the posterior inclusion probability (PIP) for each creatinine-adjusted VOC-EB, as well as the overall effect of VOC-EB mixtures on Ln CAP and Ln LSM, with adjustments for potential confounders. The final model utilized 10,000 iterations in a Markov Chain Monte Carlo (MCMC) sampler (31). A PIP threshold of 0.5 is commonly applied to determine the significance of the VOC-EBs. Estimates at any percentile, where the 95% confidence intervals excluded zero relative to the 50th percentile, were considered statistically significant (25, 34).

Data analysis was performed using R version 4.2.3, and BKMR analyses were conducted using the “bkmr” package. A two-tailed *p*-value of less than 0.05 was considered to indicate statistical significance.

## 3 Results

### 3.1 Baseline characteristics of study population

A total of 1,854 participants were included in the study. Descriptive characteristics of the study population are detailed in Table 1. Among the participants, 44.0% (816/1,854) had liver steatosis and 10.0% (186/1,854) had liver fibrosis. Participants with liver steatosis and fibrosis were predominantly male, tended to be older, of Mexican American ethnicity, past smokers, and had conditions such as diabetes, overweight/obesity, hypertension, and lower levels of physical activity. The distribution, parent compounds, and detectable rates of 20 VOC metabolites among the 1,854 participants are detailed in Supplementary Table S1. Strong correlations were observed between HPMMA and ΣUACLM (*r* = 0.92), HPMMA and IPMA3 (*r* = 0.92), HPMMA and ΣUACLNM (*r* = 0.86), ΣUACLM and ΣUACLNM (*r* = 0.84), IPMA3 and ΣUACLNM (*r* = 0.81), and IPMA3 and ΣUACLM (*r* = 0.85), as shown in Supplementary Figure S1. The concentrations of creatinine-adjusted urinary VOC-EBs from four consecutive NHANES cycles are shown in Supplementary Figure S2. A continuous increasing trend in the concentrations of creatinine-adjusted urinary ΣUBUM, ΣUACLM, ΣUAAM, ΣUSEBM, and BPMA was observed.

**Table 1:** Descriptive characteristics of participants stratified by the presence of liver steatosis (CAP score ≥ 274 dB/m) and fibrosis (LSM score ≥ 8 kPa).

Characteristics	Total (*n* = 1,854)	Stestosis (CAP score ≥ 274 dB/m)			Fibrosis (LSM ≥ 8 k.Pa)		
		No (*n* = 1,038)	Yes (*n* = 816)	*p*-value	No (*n* = 1,668)	Yes (*n* = 186)	*p*-value
Gender (%)				<0.001			0.002
Male	922 (49.73)	474 (45.66)	448 (54.90)		809 (48.50)	113 (60.75)	
Female	932 (50.27)	564 (54.34)	368 (45.10)		859 (51.50)	73 (39.25)	
Age (years)	50.28 (16.76)	48.28 (17.34)	52.83 (15.63)	<0.001	49.76 (16.84)	54.99 (15.31)	<0.001
Race-ethnicity (%)				<0.001			0.137
Mexican American	217 (11.70)	85 (8.19)	132 (16.18)		192 (11.51)	25 (13.44)	
Hispanic	174 (9.39)	98 (9.44)	76 (9.31)		152 (9.23)	20 (10.75)	
Non-Hispanic White	657 (35.44)	353 (34.01)	304 (37.26)		587 (35.19)	70 (37.63)	
Non-Hispanic Black	491 (26.46)	313 (30.15)	178 (21.81)		439 (26.32)	52 (27.96)	
Others	315 (16.99)	189 (18.21)	126 (15.44)		296 (17.75)	19 (10.22)	
Education (%)				0.083			0.074
High school or less	763 (41.15)	407 (39.21)	356 (43.63)		677 (40.59)	86 (46.24)	
College	616 (33.23)	347 (33.43)	269 (32.97)		551 (33.03)	65 (34.95)	
Graduate or higher	475 (25.62)	284 (27.36)	191 (23.41)		440 (26.38)	35 (18.82)	
Smokers (%)				0.042			0.584
Never	1,058 (57.07)	594 (57.23)	464 (56.86)		954 (57.19)	104 (55.91)	
Former	465 (25.08)	242 (23.31)	223 (27.33)		413 (24.76)	52 (27.96)	
Current	331 (17.85)	202 (19.46)	129 (15.81)		301 (18.05)	30 (16.13)	
Alcohol use (%)				0.292			0.197
Never drinkers	492 (26.54)	276 (26.59)	216 (26.47)		434 (26.02)	58 (31.18)	
Low-moderate drinkers	687 (37.06)	370 (35.65)	317 (38.65)		617 (36.99)	70 (37.63)	
Heavy drinkers	675 (36.41)	392 (37.76)	283 (34.68)		617 (36.99)	58 (31.18)	
Diabetes (%)				<0.001			<0.001
No	1,611 (86.89)	963 (92.77)	648 (79.41)		1,487 (89.15)	124 (66.67)	
Yes	243 (13.11)	75 (7.23)	168 (20.59)		181 (10.85)	62 (33.33)	
Overweight/obesity (%)				<0.001			<0.001
No	481 (25.94)	416 (40.08)	65 (7.97)		463 (27.76)	18 (9.68)	
Yes	1,373 (74.06)	622 (59.92)	751 (92.03)		1,205 (72.24)	168 (90.32)	
Hypertension (%)				<0.001			<0.001
No	1,171 (63.16)	721 (69.46)	450 (55.15)		1,086 (65.11)	85 (45.7)	
Yes	683 (36.84)	317 (30.54)	366 (44.85)		582 (34.89)	101 (54.3)	
Physical activity (%)				<0.001			0.030
No	947 (51.08)	486 (46.82)	461 (56.50)		838 (50.24)	109 (58.60)	
Yes	907 (48.92)	552 (53.18)	355 (43.51)		830 (49.76)	77 (41.40)	
PIR (%)				0.382			0.822
<1	318 (17.15)	171 (16.47)	147 (18.01)		285 (17.09)	33 (17.74)	
≥1	1,536 (82.85)	867 (83.53)	669 (81.99)		1,383 (82.91)	153 (82.26)	

Data are based on the National Health and Nutrition Examination Survey (NHANES) from 2017 to 2020, with a total sample size of 1,854 participants.

### 3.2 Association of single urinary VOC-EBs with liver steatosis and fibrosis

**Figure 2:**
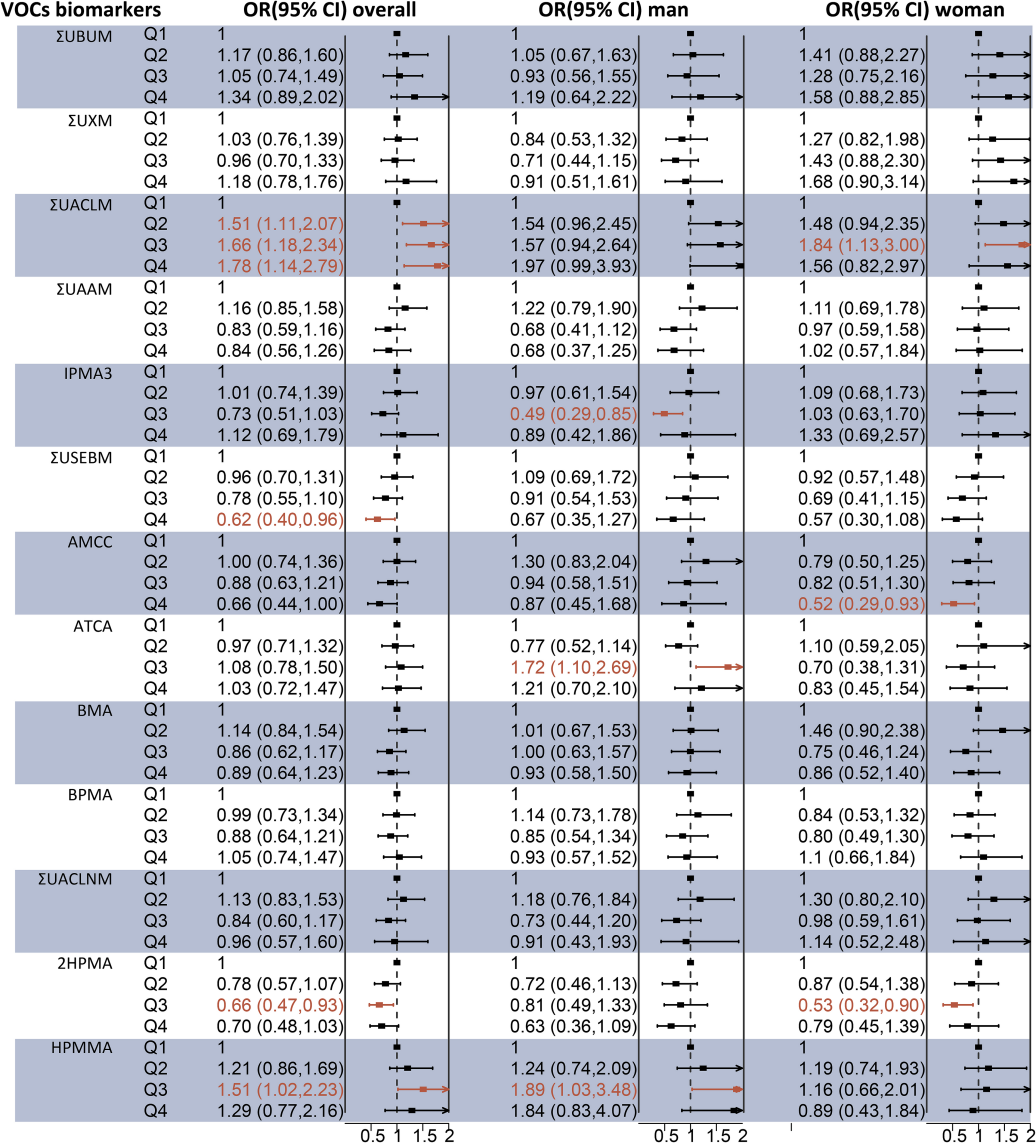
Association between individual urinary VOC-EB concentrations and the risk of liver steatosis, analyzed overall and stratified by sex, using multivariable logistic regression models. All VOC-EB concentrations were normalized to urinary creatinine levels (units: μg/g creatinine). Results are presented as odds ratios (OR) with 95% confidence intervals (CI). The regression model was adjusted for age, gender, race, obesity status, diabetes status, hypertension status, smoking status, alcohol use, physical activity, education level, and poverty income ratio (PIR). A bold segment indicates a *p*-value < 0.05.

Binary logistic regression models were employed to assess the individual effects of each urinary VOC-EB on hepatic steatosis and fibrosis. As depicted in Figure 2, after adjusting for covariates, a significant positive association was observed between ΣUACLM and liver steatosis in a dose–response pattern (p-trend < 0.05). Additionally, HPMMA was positively associated with liver steatosis in the third quartile compared to the first quartile [OR 1.51 (95% CI 1.02–2.23)]. Meanwhile, negative associations with liver steatosis were observed for ΣUSEBM and 2HPMA. The adjusted OR for liver steatosis was 0.62 (95% CI 0.40–0.96) among participants in the highest urinary ΣUSEBM quartile. The adjusted OR for urinary 2HPMA with liver steatosis was 0.66 (95% CI 0.47–0.93) in the Q3 compared to Q1. Notable differences were observed in the sex-stratified analysis. For example, ΣUACLM and ΣUSEBM exhibited similar correlations in both men and women. However, high concentrations of ACTA and HPMMA were positively associated with liver steatosis, while high concentrations of IPMA3 were negatively associated with liver steatosis in men. For women, high concentrations of AMCC and 2HPMA were negatively associated with liver steatosis (Figure 2).

**Figure 3:**
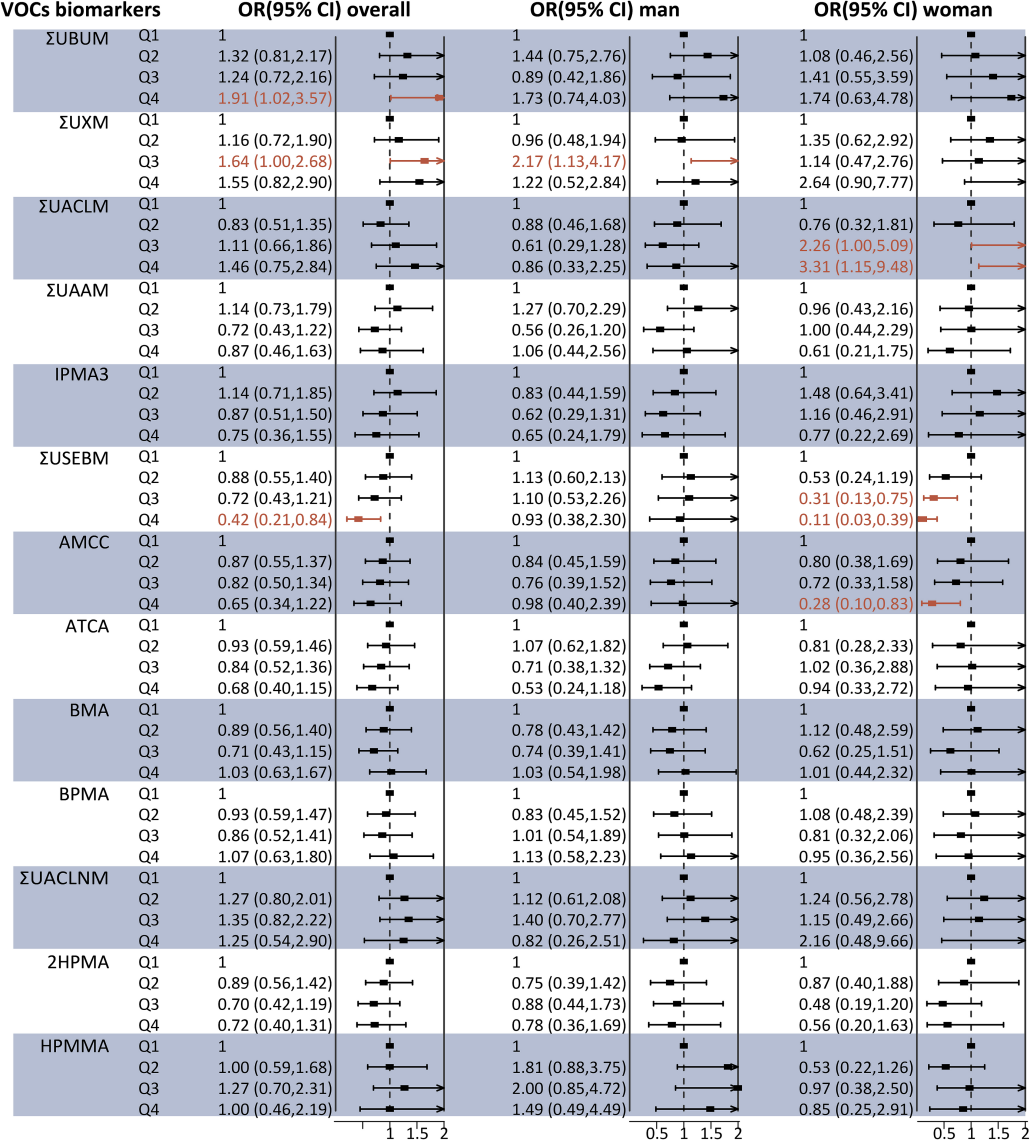
Association between individual urinary VOC-EB concentrations and the risk of liver fibrosis, analyzed overall and stratified by sex, using multivariable logistic regression models. All VOC-EB concentrations were normalized to urinary creatinine levels (units: μg/g creatinine). Results are presented as odds ratios (OR) with 95% confidence intervals (CI). The model was adjusted for age, gender, race, obesity status, diabetes status, hypertension status, smoking status, alcohol use, physical activity, education level, and poverty income ratio (PIR). Segments highlighted in bold indicate a *p*-value < 0.05.

Regarding liver fibrosis, as illustrated in Figure 3, binary logistic regression analysis indicated that participants in the highest quartile of ΣUBUM (Q4 vs. Q1: OR = 1.91, 95% CI: 1.02–3.57) and ΣUXM (Q3 vs. Q1: OR = 1.64, 95% CI: 1.00–2.68) exhibited a higher prevalence of liver fibrosis compared to those in the first quartile. Additionally, a negative association was observed between ΣUSEBM and liver fibrosis in the third quartile compared to the first quartile [OR 0.42 (95% CI 0.21–0.84)]. However, the analysis stratified by sex revealed several inconsistencies. High concentrations of ΣUXM were positively associated with liver fibrosis in men, a finding consistent with the overall population. Meanwhile, high concentrations of ΣUSEBM and AMCC were negatively associated with liver fibrosis, and high concentrations of ΣUACLM were positively associated with liver fibrosis in women. Associations of urinary VOC-EBs mixture exposure with Ln CAP and Ln LSM.

**Figure 4:**
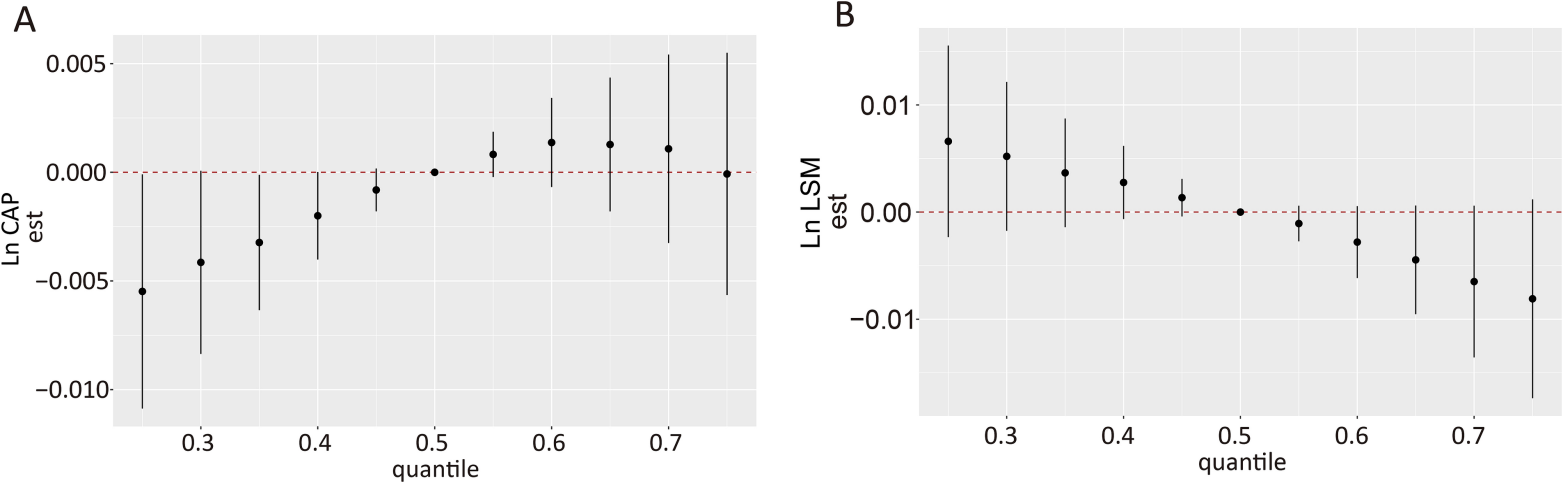
Overall association of VOC-EB mixtures with Ln CAP and Ln LSM using the Bayesian Kernel Machine Regression (BKMR) model. **(A)** Association of the urinary VOC-EB mixture with Ln CAP. **(B)** Association of the urinary VOC-EB mixture with Ln LSM. Both panels depict results adjusted for age, gender, race, obesity status, diabetes status, hypertension status, smoking status, alcohol use, physical activity, education level, and poverty income ratio (PIR).

Ln-transformed CAP, LSM, and concentrations of each VOC-EB were treated as continuous variables, and the BKMR model was fitted to assess their joint effects on CAP and LSM. Although the confidence intervals were broad, a significant decrease in Ln CAP was observed at the 35th percentile or below compared to the 50th percentile, indicating a significant positive association (Figure 4A). A decreasing trend was observed in the Ln LSM values, although these did not reach statistical significance (Figure 4B). The PIPs of ΣUACLM and ΣUACLNM for Ln CAP exceeded 0.5 (Supplementary Table S2), suggesting these VOC-EBs largely contributed to the observed increase in Ln CAP. Significant associations for ΣUACLM and ΣUACLNM with Ln CAP were observed regardless of other VOC-EBs being fixed at their 25th, 50th, or 75th percentiles (Supplementary Figure S3A), similar to BPMA and ΣUBUM with Ln LSM (Supplementary Figure S3B).

**Figure 5:**
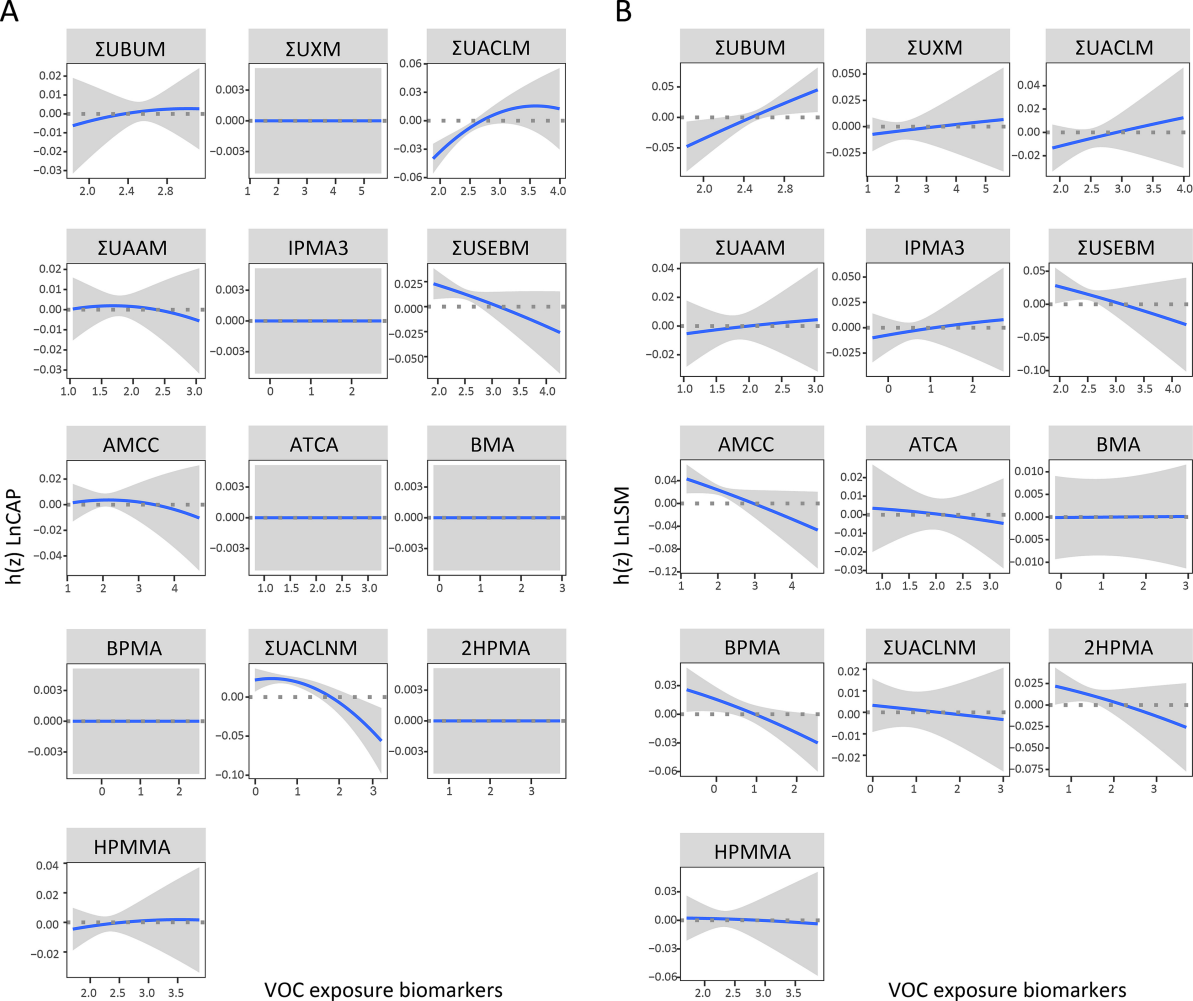
Univariate exposure-response functions displaying the associations of selected VOC-EBs with Ln CAP **(A)** and Ln LSM **(B)**. Each function is plotted with 95% confidence intervals (CIs) and analyzes the relationship while fixing the concentrations of other chemicals at their median values. The BKMR models used for these analyses were adjusted for age, gender, race, obesity status, diabetes status, hypertension status, smoking status, alcohol use, physical activity, education level, and poverty income ratio (PIR).

The dose–response relationships of the 13 VOC-EBs were illustrated after adjusting for covariates with other VOC-EBs set at their median levels (Figure 5). Positive exposure-response relationships were observed between ΣUACLM and Ln CAP, whereas negative associations were noted for ΣUSEBM and ΣUACLNM (Figure 5A). Variable patterns were noted in Ln LSM: ΣUBUM demonstrated a positive relationship, while ΣUSEBM, AMCC, BPMA, and 2HPMA showed inverse relationships (Figure 5B).

## 4 Discussion

To our knowledge, this study is the first to characterize the distributions of 20 urinary VOC metabolites in the general population from 2011 to 2020 and to assess their associations with hepatic steatosis and liver fibrosis using VCTE and diverse statistical methods. The results demonstrated a continuous increasing trend in several urinary VOC-EBs from 2011 to 2020. On one hand, from 2017 to 2020, multivariate logistic regression indicated that several urinary VOC-EBs were significantly associated with an increased risk of hepatic steatosis and liver fibrosis, while several urinary VOC-EBs significant negative associations. Furthermore, the relationships of VOC-EBs with liver steatosis and fibrosis in men and women were found to be sporadic and inconsistent. On the other hand, BKMR analysis revealed that overall mixed exposure was significantly positively associated with Ln CAP. The univariate exposure-response function identified associations between several urinary VOC-EBs and the risk of hepatic steatosis and liver fibrosis. Both multivariate logistic regression and BKMR analysis found that ΣUACLM (acrolein metabolites) was positively associated with hepatic steatosis, ΣUBUM (1,3-butadiene metabolites) with liver fibrosis, and ΣUSEBM (ethylbenzene metabolites) negatively with both conditions. These findings underscore the role of environmental VOC exposure in the development of hepatic steatosis and fibrosis.

VOCs originate from various sources, with road traffic emissions constituting the largest share before 2010. The replacement of older vehicles and the gradual reduction in gasoline consumption contributed to a rapid decline in VOC emissions until 2010 (35). From 2010 onwards, the use of solvents is believed to have surpassed road traffic as the primary source of VOCs, stabilizing pollution levels (36). In this study, we observed a trend of continuous increase in the urinary concentrations of ΣUBUM, ΣUACLM, ΣUAAM, ΣUSEBM, and BPMA, derived from parent compounds such as 1,3-butadiene, acrolein, acrylamide, styrene, ethylbenzene, and 1-bromopropane. Most governments have not yet to implement regulations on the use of solvents (35), which may partially explain these findings. In fact, the US EPA has found that the levels of about a dozen VOCs are 2 to 5 times higher inside homes than outside, whether in rural or highly industrial areas (37). Additionally, VOCs detected in indoor air and tap water are often as numerous and varied as those found outdoors (10). Clearly, exposure to these ambient VOCs is inevitable in everyday life. Further research is required to confirm these findings, and increased attention should be directed toward the rising exposure to VOCs among the general population.

While epidemiological studies on the association between combined VOC exposures and hepatic steatosis or fibrosis are sporadic, individual VOC exposures have been implicated in liver injury within the general population. A cross-sectional study within the Canadian population demonstrated that blood concentrations of xylene, styrene, and toluene were associated with elevated levels of ALP and AST (24). An earlier study involving 663 US adults showed a positive association between ALP levels and urinary exposure biomarkers for acrolein, xylene, and 1,3-butadiene (23). Furthermore, a recent study of the US general adult population reported significant correlations between urinary metabolites of acrolein, 1,3-butadiene, and xylene, and NAFLD as defined by the USFLI (25). However, levels of these serum liver enzymes may be comparable in patients with or without liver steatosis (38), and these biomarkers are influenced by comorbid conditions, potentially reducing their sensitivity in defining steatosis (26, 27). According to guidelines from the American Gastroenterological Association, VCTE is preferred for the precise quantification of liver fat (CAP) and fibrosis (LSM) (39, 40). We initially discovered significant associations between urinary ΣUACLM (an exposure biomarker for acrolein) and hepatic steatosis, and between urinary ΣUBUM and ΣUXM (exposure biomarkers for 1,3-butadiene and xylene, respectively) and liver fibrosis, as diagnosed by VCTE, aligning with findings from previous studies. Interestingly, urinary ATCA and HPMMA was positively associated with liver steatosis in men, but not in women, while ΣUACLM was positively associated with liver steatosis and fibrosis in women, but not in men. The sex differences in the health hazards of urinary VOCs have also been reported. A previous study showed that sex significantly interacted with ΣUAAM in influencing the liver steatosis biomarker (25). Another study indicated that increasing levels of VOCs were associated with increases in C-reactive protein for women, but not for men (24). The hypothesis explains the sex-based differential susceptibility, including sex hormones, anatomical differences, gut microbiota, and epigenetic effects (33). Furthermore, obesity, with its increasing prevalence worldwide, has been recognized as a significant contributor to VOC toxicity and a key driver of NAFLD onset and progression, including fibrosis. Recent studies have demonstrated the association between VOC exposure and obesity in the general U.S. population (41), underscoring the complex interaction between environmental pollutants and metabolic disorders. Furthermore, high body mass index (BMI), a major risk factor for NAFLD, frequently coexists with sarcopenia, a condition characterized by loss of muscle mass and strength (42). Sarcopenia is prevalent in advanced liver disease and is closely associated with obesity, forming a “sarcopenic obesity” phenotype that exacerbates liver injury and fibrosis progression. Given the role of obesity in liver steatosis and fibrosis, and its association with sarcopenia, it is plausible that VOC exposure contributes to both conditions. Sarcopenia and obesity not only exacerbate NAFLD progression but also amplify susceptibility to environmental toxins such as VOCs, leading to a compounded health burden. Future studies are warranted to explore the mechanistic pathways underlying the interaction between VOC exposure, obesity, and sarcopenia.

Traditional generalized linear regression models, including multivariable linear and logistic regression, typically offer straightforward relationships between individual VOCs and health outcomes (43, 44). However, these models often overlook mixed environmental exposures, joint effects, and their nonlinear interactions, potentially leading to false negative or positive results (34, 43, 45). Moreover, a strong correlation among several urinary VOC-EBs was detected in our study, which can distort the outcomes of generalized linear regression models (46). Thus, we utilized the BKMR model, a recently developed nonparametric statistical method, to analyze the joint effects of VOC-EBs on liver health. This novel mixture modeling approach accommodates a range of VOC-EBs, even those with high correlations (31). Furthermore, BKMR analysis tests the overall mixture effect and captures nonlinear exposure-response relationships, with other chemicals fixed at specified levels. In our analysis, a significant positive joint effect of the VOC-EBs mixture on Ln CAP was observed, particularly when urinary VOC-EB concentrations were below the 35th percentile. This finding suggests that VOC-EBs may be linked to the severity of liver steatosis. The PIPs of ΣUACL and ΣUACLNM for CAP exceeded 0.5, indicating that these VOC-EBs significantly contributed to the association with Ln CAP. A previous study demonstrated that a mixture of VOC-EBs was positively associated with liver steatosis as defined by USFLI, although the results differed when defined by HSI (25). This discrepancy may stem from the unreliability of serum biomarkers in predicting liver injury, whereas liver VCTE is likely more sensitive (38–40). Additionally, no associations were found between the mixture of VOC-EBs and LSM. A recent study found that a mixture of VOC-EBs was associated with liver fibrosis as defined by the Hepamet Fibrosis Score (HFS), but not when using the Non-Alcoholic Fatty Liver Disease Fibrosis Score (NFS) (25). Indeed, some studies suggest that VOC exposure may be linked to liver fibrosis, as smoking—a known contributor to advanced liver fibrosis—typically results in higher urinary VOC metabolite levels in smokers than in nonsmokers (23, 47, 48). The association between cigarette smoking and the risk of sarcopenia also warrants attention (11–13). Sarcopenia, common across various diseases, is one of the most frequent complications in advanced liver disease. This inconsistency could be attributed to low levels of VOC exposure in the general population and the adjustment for smoking as a confounder, considering that smoking significantly contributes to urinary VOC levels and is independently associated with sarcopenia. Furthermore, traditional risk assessment procedures, based on single chemical evaluations, do not align with the characteristics of low-level, multiple chemical exposures typical of modern life, thus overlooking the cocktail effect of mixtures, which can underestimate the health risks of pollutants. Mixtures at concentrations that individually do not cause observable adverse effects can produce harmful effects, as reviewed elsewhere (49). A possible explanation is that multiple molecular pathways can be affected by the same chemical, often exhibiting nonlinear dose–response relationships, or different pathways are affected by chemicals at various doses (50). Further research with larger sample sizes is necessary to clarify this association.

The BKMR analysis also allows for the identification of exposure-response relationships with other chemicals held at fixed levels. In our analysis, ΣUACLM (exposure biomarker for acrolein) demonstrated a positive association with CAP, consistent with the findings from individual VOC analyses, thus reinforcing this result. Furthermore, our study is the first to report that ΣUBUM (exposure biomarker for 1,3-butadiene) displayed a linear correlation with liver fibrosis, a finding more pronounced than in individual VOC analyses. Although associations between 1,3-butadiene and liver fibrosis have not been extensively studied, the liver is considered the primary site of 1,3-butadiene-induced carcinogenesis (51). DNA damage, the primary toxic effect of 1,3-butadiene in hepatocytes (52), also plays a crucial role in the pathogenesis of liver fibrosis (53). Additionally, ΣUSEBM and ΣUACLNM showed negative associations with CAP. Meanwhile, ΣUSEBM and AMCC were negatively associated with LSM, especially in the lowest concentration. These results contradict previous findings that utilized liver injury markers (20, 54, 55). Several explanations are possible for these results. First, given the lack of significant associations in individual analyses, this negative association could be due to complex antagonistic interactions among VOCs. The interactions are common among VOCs because VOCs are mostly metabolized by CYP450 enzymes in liver (17, 18, 56). Another explanation might be that, unlike high-dose occupational VOC exposure, which is positively correlated with liver injury (20, 54), generally VOCs exposure may not necessarily exert a same effect. Nevertheless, caution should be exercised in interpreting the results, and the above hypothetical explanations need to be further validated in future studies.

Although the biological mechanisms underlying the hepatotoxicity of VOCs have not been fully elucidated, evidence from numerous animal studies supports our findings with plausible molecular mechanisms. Oxidative stress is a common hepatotoxic effect induced by VOCs. Most VOCs are metabolized by CYP450 enzymes primarily during biotransformation, forming active electrophilic intermediates (17). These intermediates then conjugate with glutathione (57), the most abundant *in vivo* antioxidant, thereby indirectly aggravating oxidative stress (58). Oxidative stress is considered a potential mechanism for the development of liver steatosis and fibrosis (53). The inflammatory response plays an integral role in the progression of liver fibrosis from liver steatosis. Accumulation of VOCs such as acrolein in the liver can induce neutrophil recruitment and activation, leading to the formation of neutrophil extracellular traps (59). Exposure to 1,3-butadiene was found to upregulate genes involved in oxidative and inflammatory responses in the lungs of mice (60). Furthermore, acrolein can significantly increase the expression of endoplasmic reticulum (ER) stress markers in hepatocytes (61), Furthermore, acrolein can significantly increase the expression of endoplasmic reticulum (ER) stress markers in hepatocytes (62). However, the causal role of VOCs in the initiation and progression of liver diseases, as discussed in this study, warrants further investigation.

There are several limitations of our study. First, the cross-sectional design of this study precludes definitive conclusions about the causal relationships between urinary VOC mixtures and liver steatosis or hepatic fibrosis. Case–control or cohort studies are needed to address this methodological limitation. Second, although VCTE offers many benefits, it is not the gold standard for diagnosing liver conditions. Liver biopsy remains the gold standard for diagnosing liver steatosis and fibrosis, but recruiting sufficient participants from the general population is challenging. In this study, VCTE was performed by trained NHANES health technicians to maximize the accuracy of the results. Third, while our models adjusted for many confounders, the potential for unmeasured confounding remains. Specifically, data on genetic susceptibility, drug usage and dosages, and treatment adherence were lacking. Finally, as this study was conducted among U.S. adults, the generalizability of our findings to other populations is uncertain.

## 5 Conclusion

In conclusion, our study suggests that exposure to VOC mixtures increases the prevalence of liver steatosis among U.S. adults. Acrolein may play a significant role in the association between VOC mixture exposure and liver steatosis, while 1,3-butadiene may be linked to an increased risk of liver fibrosis. These findings underscore the significant role of environmental VOC mixture exposure in the development of liver steatosis and hepatic fibrosis. Further prospective cohort studies and mechanistic research are required to validate these conclusions.

## Data Availability

Publicly available datasets were analyzed in this study. This data can be found here: https://wwwn.cdc.gov/nchs/nhanes/Default.aspx.
