# Supplementary material for: Association of exposure to multiple volatile organic compounds with ultrasound-defined hepatic steatosis and fibrosis in the adult US population: NHANES 2017–2020
Source: Front Public Health. 2025 Jan 17;12:1437519. doi: 10.3389/fpubh.2024.1437519 (PMC11782259; doi:10.3389/fpubh.2024.1437519)
Supplement: Supplementary file 1 [file Table_1.DOCX]

Supplementary Material

# Supplementary Figures and Tables

## Supplementary Figures


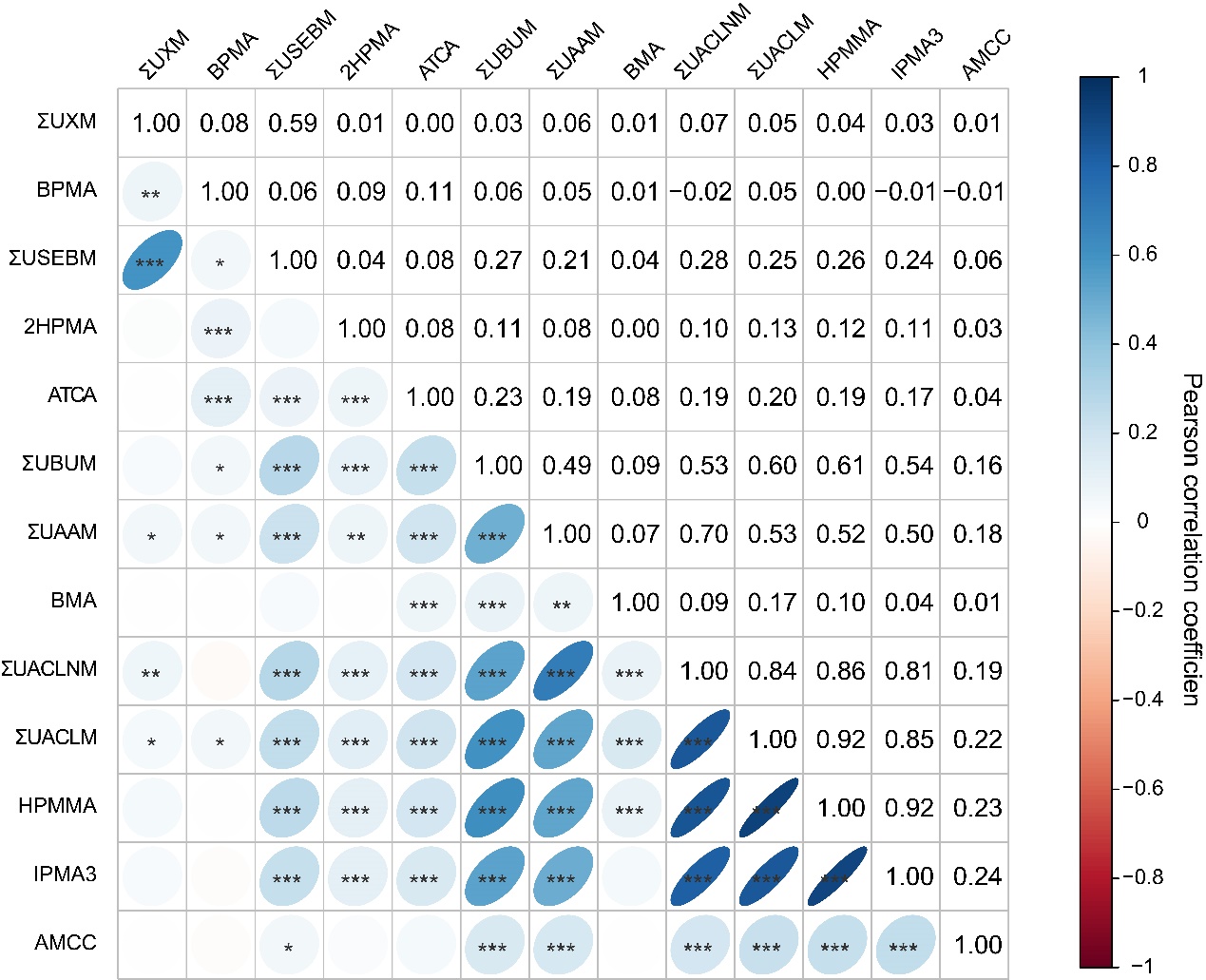


**Supplementary Figure 1.** Pairwise Pearson correlations among urinary VOC-EBs (creatinine adjusted) in the study population from the National Health and Nutrition Examination Survey (NHANES) 2017–2020 (N = 1854). Blue ellipses in the grid represent positive correlations, while red ellipses indicate negative correlations. Each cell displays the Pearson correlation coefficient. Significance levels are denoted as: *, p < 0.05; **, p < 0.01; ***, p < 0.001.


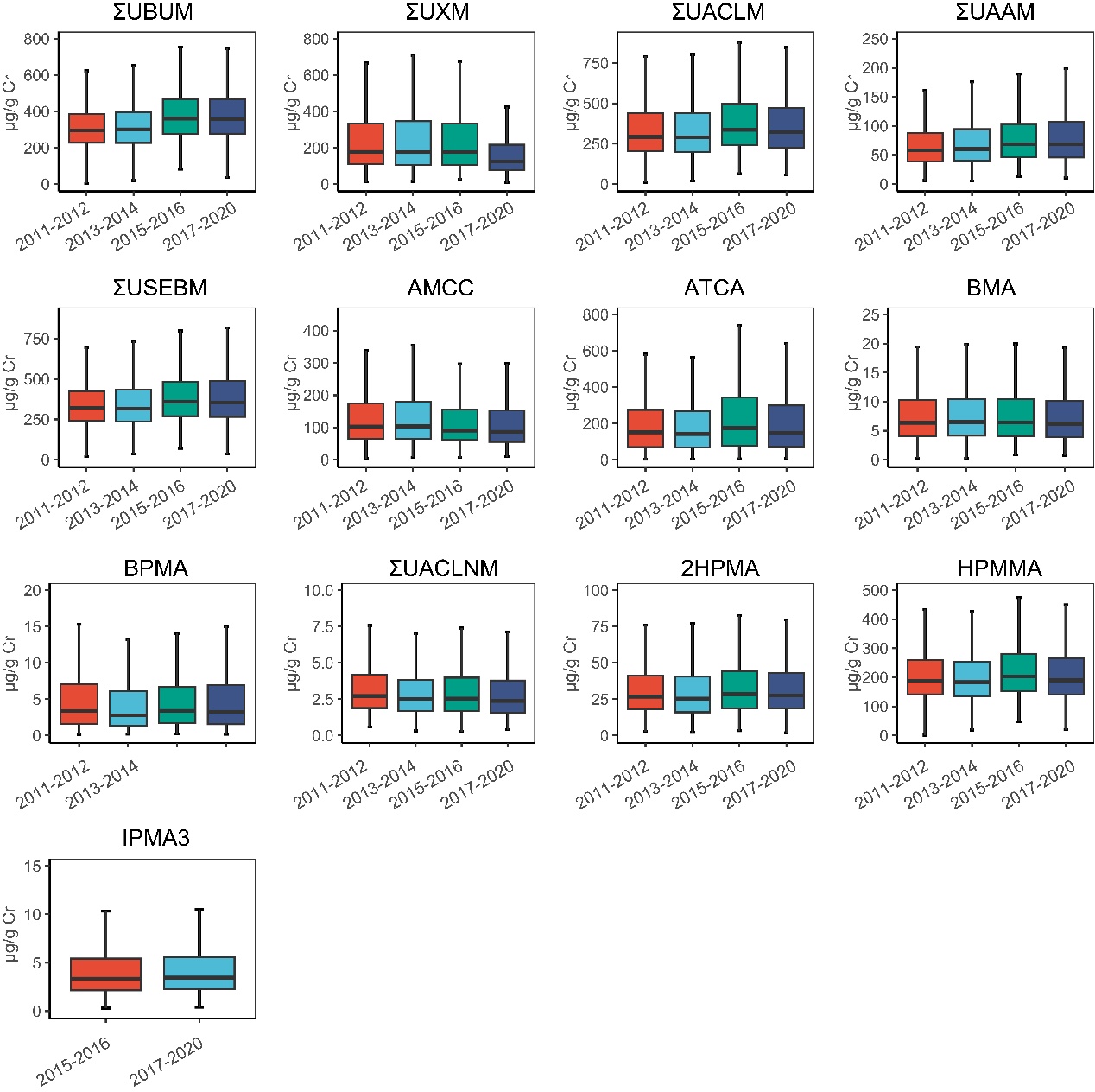


**Supplementary Figure 2.** Box plot depicting the trends of urinary VOC-EBs (creatinine adjusted) across four NHANES cycles from 2011 to 2020. Each box represents the interquartile range (IQR), while the error bars indicate the minimum and maximum values observed in the population.


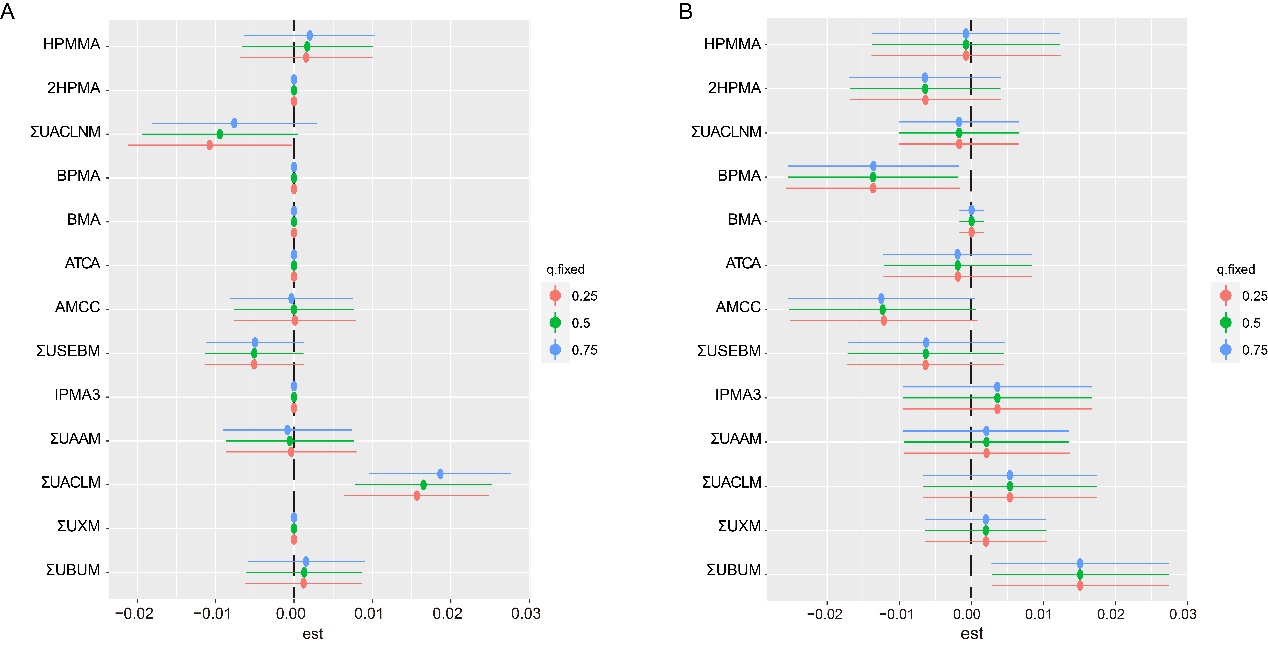


**Supplementary Figure 3.** Associations of single VOC-EBs with Ln CAP and Ln LSM using the Bayesian Kernel Machine Regression (BKMR) model. (A) Displays the impact of urinary VOC-EB mixtures on Ln CAP. (B) Illustrates the influence of urinary VOC-EB mixtures on Ln LSM. Both models were adjusted for age, gender, race, obesity status, diabetes status, hypertension status, smoking status, alcohol use, physical activity, education level, and poverty income ratio (PIR)

## Supplementary Table

**Supplementary Table 1.** List of the 20 volatile organic compounds (VOC) metabolites, along with their parent compounds, distributions, and detectable rates, as measured in the National Health and Nutrition Examination Survey (NHANES) from 2017 to 2020

| Parent Compound | VOC Metabolites | Common Abbr. | Median (25percentile-75percentile, μg/g cr) | Detectable rates |
| --- | --- | --- | --- | --- |
| 1,3-butadiene (ΣUBUM) | N-ace-S-(3,4-dihidxybutl)-L-cys | DHBMA | 355.56 (276.71, 473.00) | 100.00 |
|  | N-A-S-(4-hydrxy-2-butenyl)-L-cys | MHBMA3 | 4.69 (2.86, 8.57) | 97.46 |
| Xylene (ΣUXM) | 2-methylhippuric acid | 2MHA | 22.82 (12.51, 50.57) | 88.28 |
|  | 3-methipurc acd + 4-methipurc acd | 3MHA +4MHA | 109.73 (66.79, 224.37) | 99.33 |
| Acrolein (ΣUACLM) | N-ace-S-(2-carbxyethyl)-L-cys | CEMA | 97.17 (60.95, 155.20) | 98.68 |
|  | N-ace-S-(3-hydroxypropyl)-L-cys | 3HPMA | 260.54 (163.00, 468.50) | 99.70 |
| Acrylamide (ΣUAAM) | N-ace-S-(2-carbamoylethyl)-L-cys | AAMA | 57.88 (36.54, 97.17) | 99.93 |
|  | N-ac-S-(2-carbmo-2-hydxel)-L-cys | GAMA | 10.97 (7.07, 17.94) | 41.43 |
| Styrene, ethylbenzene (ΣUSEBM) | mandelic acid | MA | 136.45 (96.17, 200.92) | 98.91 |
|  | phenylglyoxylic acid | PGA | 226.76 (164.39, 316.67) | 99.86 |
| Acrylonitrile (ΣUACLNM) | N- ace -S-(1-cyano-2-hydroxyethyl)-L-cys | CHEMA | 2.39 (1.35, 5.58) | 18.43 |
|  | N- ace -S-(2-cyanoethyl)-L-cys | CEMA | 1.53 (0.89, 3.60) | 80.95 |
|  | N-ace-S-(2-hydroxyethyl)-L-cys | 2HEMA | 1.10 (0.59, 2.12) | 46.61 |
| Isoprene | N-ace-S-(4-hydroxy-2-methyl-2-buten-1-yl)-L-cys | IPMA3 | 3.96 (2.39, 7.64) | 82.45 |
| N,N-dimethylformamide | N-ace-S-(N-methlcarbamoyl)-L-cys | AMCC | 92.61 (57.26, 177.92) | 99.35 |
| Cyanide | 2-aminothiazoline-4-carboxylic acid | ATCA | 166.94 (75.82, 365.81) | 90.27 |
| Toluene | N- ace -S-(benzyl)-L-cys | BMA | 6.64 (4.05, 11.71) | 99.72 |
| 1-bromopropane | N-ace-S-(n-propyl)-L-cys | BPMA | 4.13 (1.76, 10.55) | 73.92 |
| Propylene oxide | N-ace-S-(2-hydroxypropyl)-L-cys | 2HPMA | 29.88 (19.74, 52.07) | 94.73 |
| Crotonaldehyde | N-ace-S-(3-hydrxprpl-1-metl)-L-cys | HPMMA | 214.00 (149.26, 348.45) | 100.00 |

**Supplementary Table 2.** Posterior inclusion probabilities (PIPs) for inclusion each VOC-EBs into Ln CAP and Ln LSM components models, using Bayesian kernel machine regression (BKMR) model

| VOC-EBs | Ln CAP | Ln LSM |
| --- | --- | --- |
| ΣUBUM | 0.067 | 0.329 |
| ΣUXM | 0.000 | 0.010 |
| ΣUACLM | 0.586 | 0.065 |
| ΣUAAM | 0.114 | 0.060 |
| IPMA3 | 0.000 | 0.056 |
| ΣUSEBM | 0.033 | 0.115 |
| AMCC | 0.049 | 0.108 |
| ATCA | 0.000 | 0.038 |
| BMA | 0.000 | 0.000 |
| BPMA | 0.000 | 0.171 |
| ΣUACLNM | 0.518 | 0.002 |
| 2HPMA | 0.000 | 0.113 |
| HPMMA | 0.046 | 0.065 |
